# Foot-and-Mouth Disease Virus Serotype SAT 3 in Long-Horned Ankole Calf, Uganda

**DOI:** 10.3201/eid2101.140995

**Published:** 2015-01

**Authors:** Moses Tefula Dhikusooka, Kirsten Tjørnehøj, Chrisostom Ayebazibwe, Alice Namatovu, Simon Ruhweza, Hans Redlef Siegismund, Sabenzia Nabalayo Wekesa, Preben Normann, Graham J. Belsham

**Affiliations:** Ministry of Agriculture Animal Industry and Fisheries, Entebbe, Uganda (M.T. Dhikusooka, C. Ayebazibwe, A. Namatovu, S. Ruhweza);; Technical University of Denmark, Kalvehave, Denmark (K. Tjørnehøj, P. Normann, G.J. Belsham);; Makerere University, Kampala, Uganda (A. Namatovu, S.N. Wekesa);; University of Copenhagen, Copenhagen, Denmark (H.R. Siegismund);; Ministry of Livestock Development, Nairobi, Kenya (S.N. Wekesa)

**Keywords:** Foot-and-mouth disease virus, aphthovirus, picornavirus, full-genome sequence, viruses, Uganda

## Abstract

After a 16-year interval, foot-and-mouth disease virus serotype SAT 3 was isolated in 2013 from an apparently healthy long-horned Ankole calf that grazed close to buffalo in Uganda. The emergent virus strain is ≈20% different in nucleotide sequence (encoding VP1 [viral protein 1]) from its closest relatives isolated previously from buffalo in Uganda.

Foot-and-mouth disease (FMD) remains one of the most economically important diseases of livestock, costing ≈US $10 billion annually (*1*). Outbreaks occur in many countries, and normally disease-free countries can incur huge costs after incursions (e.g., the United Kingdom in 2001). The disease results from infection with FMD virus (FMDV, the prototypic aphthovirus within the *Picornaviridae* family) (*2*). Seven serotypes of FMDV are known; serotypes O and A are widely distributed, and the Southern African Territories (SAT) serotypes (1, 2, and 3) usually are restricted to Africa. Serotype Asia 1 has never circulated within Africa; serotype C has not been identified anywhere since 2005 (*2*,*3*). SAT 3 FMDV is the least well–characterized serotype; the most recent incidence of SAT 3 reported by the FMD World Reference Laboratory (Pirbright Institute, Woking, UK) was in buffalo within the Kruger National Park (South Africa) in 2006. In contrast, SAT 1 and SAT 2 FMDVs are much more common; a major incursion of SAT 2 into the Middle East occurred in 2012 (*4*), and outbreaks caused by these serotypes have occurred in many African countries (http://www.wrlfmd.org/fmd_genotyping/2013.htm).

In Uganda, FMD is endemic, and serotypes O and SAT 2 are the most common. In Uganda, SAT 3 FMDV was most recently identified in 1997 in buffalo in the Queen Elizabeth National Park (QENP) (*5*). SAT 1 and SAT 2 viruses were isolated from buffalo in QENP in 2006, and serologic test results indicated the presence of antibodies against SAT 3 virus; however, because cross-reactivity between serotypes occurs in these assays, this finding was not conclusive (*6*).

## The Study

In 2013, as part of a study of FMDV transmission between wildlife, especially African buffalo (*Syncerus caffer*), and domestic animals, 20 long-horned Ankole cattle (≈6 months of age) were introduced as sentinel animals into Nyakatonzi (Kasese District), in close proximity to the QENP. At the time they were transported, these animals, originating from another area where FMD outbreaks had not been reported for ≈10 years, had no circulating antibodies against FMDV nonstructural proteins (NSPs) (measured by using the PrioCHECK FMDV NS ELISA kit [Prionics, Schlieren-Zurich, Switzerland]). Blood and probang samples (comprising oropharyngeal scrapings and fluid) were obtained from individual animals at 2-week intervals after their entry to the farm from which they moved regularly into the QENP for pasture and water. These sentinel animals freely mixed and grazed with buffalo (observed within a few meters of each other) and other local cattle. More than 6,000 buffalo are present within the QENP.

No clinical signs of FMD were observed in the sentinel cattle, but serum samples were assayed for antibodies against FMDV NSPs (serotype independent); RNA was extracted from the probang samples and analyzed for FMDV genomes by using pan-serotypic real-time reverse transcription PCR (RT-qPCR) (*7*). FMDV RNA was clearly detected (cycle threshold 21) in the probang sample from animal no. 34 at 2 weeks after its introduction into the QENP. Antibodies against FMDV NSPs had developed in this animal (Table 1), and we detected high-titer antibodies against both FMDV SAT 1 and SAT 3 antigens using solid-phase blocking ELISAs (SPBEs) (Table 1); hence this calf simultaneously had FMDV RNA in the oropharynx and antibodies against FMDV in serum. FMDV is maintained in the oropharynx of cattle for ≈10 days after infection (*8*) and continues after viremia has resolved coincident with the production of antibodies against FMDV. Infectious FMDV (albeit at low levels) can be maintained within the oropharynx after infection for up to 3 and 5 years in “carrier” cattle and buffalo, respectively (*9*). Seroconversion against FMDV NSPs was observed in a second animal (no. 33) by 30 days after introduction to the QENP (Table 1). In the SPBEs, reactivity against SAT 1 was mainly detected in this animal. However, during the early stage of seroconversion, cross-reactivity occurs between the serotypes in these assays (K. Tjørnehøj, unpub. data).

**Table 1:** Detection of anti-FMDV in serum and FMDV RNA in probang samples from long-horned Ankole calves, Uganda, 2013*

Sampling day†	Calf no. 34							Calf no. 33					
	Anti-NSP, PI	SPBE titers‡				FMDV RNA in probang, C_t_		Anti-NSP, PI	SPBE titers†				FMDV RNA in probang, C_t_
O	SAT 1	SAT 2	SAT 3	O	SAT 1	SAT 2	SAT 3
0	22	–	–	–	–	–		35	–	–	–	–	–
14	**55**	–	160	–	80	21§		25	<10	10	10	10	–
30	**72**	–	80	–	40	–		**63**	20	160	10	10	–

*C_t_, cycle threshold; ELISA, enzyme-linked immunosorbant assay; FM**D**V, foot-and-mouth disease virus; PI, percentage inhibition values in the NSP ELISA (values >50% are considered positive and are indicated in boldface); NSP, nonstructural protein; RT-**q**PCR, real-time reverse transcription PCR; SAT, South**ern **African Territories; SPBE, solid-phase blocking ELISA; –, negative sample. †Day 0 was defined as the day the sentinel animals arrived at the farm in Nyakatonzi (Kasese District). ‡An in-house serotype-specific SPBE was used; samples positive at 1:10 dilution were titrated; values are reciprocals of** high**est positive dilution. §FMDV RNA in probang samples was assayed by RT-**q**PCR, and virus isolation was attempted from probang samples with detectable RNA and some other selected samples. The isolation of SAT 3 FMDV from the RT-**q**PCR–positive probang sample from calf no. 34 was demonstrated by serotype-specific antigen ELISA and confirmed by sequencing.

An aliquot of the probang sample from calf no. 34 was inoculated onto primary bovine thyroid cells for virus isolation. We observed cytopathic effects within 48 hours and assayed the virus harvest using serotype-specific antigen ELISAs. We observed a strong signal (optical density 1.385), indicating SAT 3 FMDV with no significant signal for other serotypes (data not shown). The presence of FMDV RNA in the cell harvest was demonstrated by using RT-qPCR (cycle threshold 11), indicating isolation and growth of FMDV; the isolate was named SAT 3 UGA/1/13. To characterize this strain, we amplified the RNA region encoding viral protein (VP) 1 by RT-PCR; the amplicon (821 bp) was sequenced with a BigDye Terminator v. 3.1 Cycle Sequencing Kit and an ABI PRISM 3730–DNA Analyzer (Applied Biosystems, Foster City, CA, USA).

**Figure 1:**
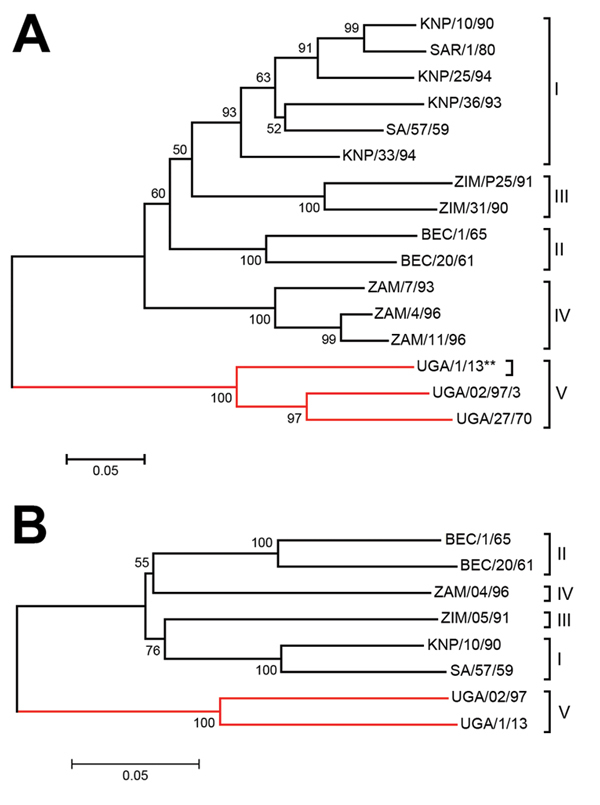
Neighbor-joining trees showing the relationships between A) the partial VP1 coding sequences (390 nt) and B) the complete P1 capsid protein coding sequence (2223 nt) from the SAT 3 FMDV UGA/1/13 isolate (marked with **) and other SAT 3 FMDVs within the indicated topotypes defined previously (*5*; http://www.wrlfmd.org/fmdv_seqs/fmdv-sat3_seq.aspx). The branches containing the Uganda viruses are indicated by the red lines. Sequences, other than for UGA/1/13, were obtained from GenBank and have been published previously (*5**,**11**–**13*). Bootstrap values are indicated. BEC, Bechuanaland (former name for Botswana); FMDV, foot-and-mouth disease virus; KNP, Kruger National Park (in South Africa); P1, precursor for the 4 capsid proteins; SAT, Southern African Territories; SAR, Republic of South Africa; UGA, Uganda; VP, viral protein; ZAM, Zambia; ZIM, Zimbabwe; I–V, topotypes. Scale bars indicate nucleotide substitutions per site.

**Figure 2:**
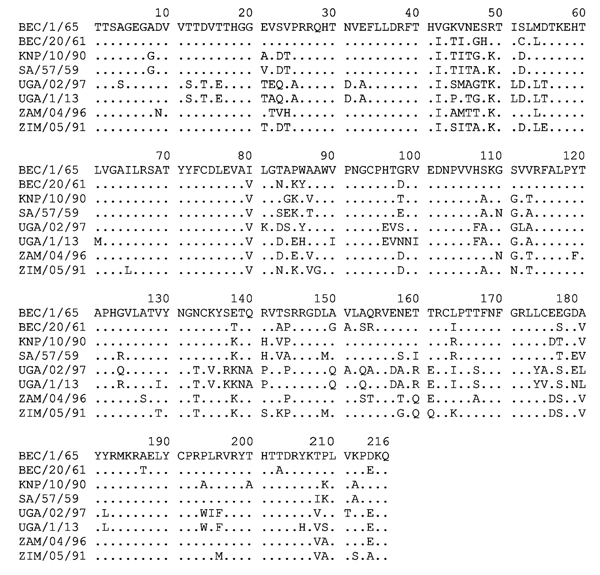
Predicted VP1 aa sequences of the 8 SAT 3 FMDVs used in the phylogenetic comparison in Figure 1, panel B. Clear similarities between the UGA/02/97 and UGA/1/13 viruses are apparent. BEC, Bechuanaland (former name for Botswana); FMDV, foot-and-mouth disease virus; KNP, Kruger National Park (in South Africa); SAR, Republic of South Africa; SAT, Southern African Territories; UGA, Uganda; VP, viral protein; ZAM, Zambia; ZIM, Zimbabwe.

Using BLAST (http://blast.st-va.ncbi.nlm.nih.gov/Blast.cgi) in MEGA5 (*10*), we found the sequence to be most closely related (81% identity) to the Ugandan SAT 3 buffalo isolate (UGA/2/97) (*5*) (Figure 1, panel A). The range of divergence was 19%–36% in this region of the genome (published VP1 coding sequences are mainly incomplete [*5*,*11*–*13*]; thus we analyzed only 390 nt from the 648 nt encoding VP1). The UGA/1/13 strain is most closely related to earlier Uganda SAT 3 viruses but is within a different lineage and is more divergent from SAT 3 viruses from southern Africa. The UGA/27/70 and UGA/2/97 viruses were assigned to topotypes V and VI, respectively (*5*); because the UGA/1/13 is ≈20% different from these strains, it could be designated as a new topotype. However, it seems better to classify these Uganda SAT 3 strains within a single topotype (V). The genome sequence (8,268 nt) of this SAT 3 virus was determined (GenBank accession no. KJ820999) from 17 overlapping amplicons (primer sequences available on request). Only 3 other full-genome sequences for SAT 3 FMDVs have been published (*13*); these are from southern African isolates obtained ≈50 years ago. These sequences are ≈80% identical to the UGA/1/13 isolate; this degree of diversity is not unexpected considering the geographic and temporal separation between them. Relationships between the SAT 3 viruses were examined for different regions of the genome (Table 2). The P1–2A capsid coding regions differ the most (≈27%). Phylogenetic relationships between known SAT 3 P1 coding sequences were determined (Figure 1, panel B). Alignment of the predicted amino acid sequences for VP1 (Figure 2) indicates high levels of variation within important antigenic regions of the virus (especially the G–H loop, residues 140–160). Neighbor-joining trees for other regions of the genome showed very similar relationships (data not shown), indicating that these viruses are monophyletic.

**Table 2:** Comparison of full-genome nucleotide sequences between different regions of the SAT 3 UGA/1/13 isolate and other SAT 3 FMDVs, 2013*

Sequence region	Length, nt	Nucleotide differences from SAT 3 UGA/1/13, %†		
		SA/57/59	BEC/20/61	BEC/1/65
5′ UTR‡ + L	1,680	24.0	24.1	22.7
P1–2A	2,282	26.6	27.1	27.1
P2	1,464	13.7	14.2	14.6
P3	2,700	13.8	13.8	13.9
3′ UTR§	118	5.9	8.4	5.0
Full genome	8,268	19.1	19.5	19.3

*BEC, Bechuanaland (former name for Botswana); FMDV, foot-and-mouth disease virus; L, leader protein coding sequence; SAT, Southern African Territories; UGA, Uganda; UTR, untranslated region. †The sequences below were published previously (*13*). ‡The region from the poly(C) tract to the first initiation codon was used for the comparison. §Excluding the poly(A) tail.

## Conclusions

Approximately 16 years after the most recent isolation of a SAT 3 FMDV from buffalo in Uganda, a new isolate (UGA/1/13) was obtained from an apparently healthy long-horned Ankole calf that was newly introduced into the QENP. To our knowledge, this is the first isolation of SAT 3 FMDV from cattle in East Africa. The VP1 coding sequence was ≈20% different from the most closely related virus strains within Uganda and up to 36% divergent from SAT 3 viruses from southern Africa. The Ugandan SAT 3 viruses should be classified within a single topotype (V), but this requires modification of the topotype definition used previously (*5*). Studies are needed to determine the consequences of infection of intensively farmed cattle by this virus.
